# Application of a Simple Microfluidic Chip Analysis Technology to Evaluate the Inhibitory Role of Protocatechuic Acid on Shear-Induced Platelet Aggregation

**DOI:** 10.1155/2021/5574413

**Published:** 2021-05-18

**Authors:** Cui He, Lihua Yu, Wenran Dan, Surong Deng, Haidong Ma, Beizhong Liu, Yuan Li

**Affiliations:** ^1^Department of Blood Transfusion of Yong-chuan Hospital, Chongqing Medical University, Chongqing 402160, China; ^2^Central Laboratory of Yong-chuan Hospital, Chongqing Medical University, Chongqing 402160, China; ^3^Key Laboratory of Laboratory Medical Diagnostics, Ministry of Education, Department of Laboratory Medicine, Chongqing Medical University, Chongqing 400016, China

## Abstract

This study aimed to develop a simple microfluidic chip analysis technology to study the inhibitory effect of protocatechuic acid on shear-induced platelet aggregation. The microfluidic chip designed in this study simulates 80% fixed narrow microchannels. This microchannel narrow model uses the finite element analysis module of the three-dimensional modeling software solidwork to analyze fluid dynamic behavior. Blood treated with protocatechuic acid at 1, 2, 4, 8, or 16 *µ*g/mL was passed through the microchannel stenosis model at a shear rate of 10,000 s^−1^. The platelet adhesion and aggregation behaviors were then measured using fluorescence microscopy and observed in real time. Simultaneously, the antiplatelet aggregation effect of protocatechuic acid was analyzed using thromboelastography and photoelectric turbidimetry. The designed stenosis model of the microfluidic chip can produce a gradient of fluid shear rate, and the gradient of fluid shear rate can induce platelet aggregation. Under this model, the degree of platelet adhesion and aggregation increased as the shear rate increased. In the experimental concentration range of 0–8 *µ*mol/mL, protocatechuic acid exerted a concentration-dependent inhibition of platelet aggregation. In contrast, thromboelastography and photoelectric turbidimetry failed to demonstrate an inhibitory effect. The microfluidic chip analysis technology developed in this study can be used to study the effect of protocatechin in inhibiting platelet aggregation induced by shear rate in vitro. This technology is simple to operate and can be used as a new type of antiplatelet aggregation analysis technology for screening studies of novel potential antiplatelet aggregation drugs.

## 1. Introduction

Adhesion and aggregation of platelets at the site of vascular injury are the basis of normal hemostasis and pathological thrombosis. Inhibition of this function poses a risk of increased bleeding [1]. Hyperfunction of platelets increases the risk of atherosclerosis, coronary artery disease, myocardial infarction, stroke, and other thrombus diseases [2] and is characterized by high morbidity, disability, and mortality [3]. Blood flow shear stress in pathological thrombosis is often abnormally high. In normal vasculature, the shear rate of the vessel wall in the vein is approximately 10–200 s^−1^ and in the aorta is approximately 300–800 s^−1^. The wall shear rate of small arteries is approximately 450–1,600 s^−1^ [4]. In the state of thrombotic disease, mild to severe arterial stenosis can reach ∼2,000–8,000 s^−1^, and the maximum wall shear rate of severely affected atherosclerotic arteries can be as high as 40,000 s^−1^ [5, 6]. Therefore, shear-induced platelet aggregation (SIPA) is a special type of platelet aggregation and the main cause of pathological thrombosis, and it is rarely seen in normal hemostasis.

Currently, research on the mechanisms underlying SIPA is mostly related to the interaction between von Willebrand factor (vWF) in the plasma and platelet membrane surface receptors, glycoprotein (GP)Ib and GPIIb/IIIa. Mechanical stimulation of the platelet membrane surface mechanical force open the ion channel Piezol; furthermore, platelet endothelial adhesion molecule 1 and receptor Plexin D1 are protein targets related to shear stress discovered in recent years, which can sense the stimulation of shear stress and induce activation of downstream proteins to affect platelet activation and aggregation. In addition, commonly used antiplatelet aggregation drugs such as aspirin and clopidogrel have been shown to be effective at inhibiting thrombosis. However, not all patients can benefit from this effect; indeed, clinical observations show that about 10% of patients with cardiovascular diseases experience reoccurrence of thromboembolic events after long-term use of aspirin [7]. An increasing number of clinical reports have reported bleeding in patients due to excessive drug doses, including increased intracranial hemorrhage and gastrointestinal bleeding. Clinically, it is therefore necessary to monitor the dosage of aspirin and clopidogrel at all times to avoid serious consequences.

In recent years, a large number of research reports have focused on the effects of Chinese herbal medicine on cardiovascular diseases. Among the traditional Chinese medicines of interest, bayberry [8] is used for cardiovascular diseases, and honey flower [9] and pomegranate seed powder [10] are used to treat type 2 diabetes. Modern pharmacological studies have shown that many traditional Chinese medicines used to promote blood circulation and remove blood stasis can improve microcirculation, blood rheology, and hemodynamics, while inhibiting platelet function and inflammation. In addition, traditional Chinese medicine has its own characteristics in the treatment of cardiovascular and cerebrovascular diseases. Anti-SIPA action of traditional Chinese medicines often has a multitarget effect and can be used to prevent and treat cardiovascular and cerebrovascular diseases through its multiple regulatory mechanisms. Currently, traditional Chinese medicine compounds with the function of inhibiting platelet aggregation include derrone [11], notoginsenoside, and ligustrazine [12]. A study has found that protocatechuic acid has an antiplatelet aggregation effect, especially for platelet aggregation induced by changes in shear-rate gradients [13]. Its mechanism of inhibiting platelet aggregation mainly involves its inhibition of the combination of GPIb and vWF, and this inhibitory effect is stronger than those of other currently used antiplatelet aggregation drugs. Simultaneously, in vitro experiments have found that protocatechuic acid can reduce bleeding. Therefore, we believe that protocatechuic acid has better therapeutic potential in terms of antiplatelet aggregation.

The microfluidic chip designed in this study provides a microchannel structure similar in size and structure to microvessels in the body, and thus, it can precisely regulate fluid dynamic behavior. Therefore, it can be used as an ideal in vitro microvascular model for analyzing blood coagulation and platelet aggregation, as well as physiological and pathological mechanisms under shear-rate regulation [14]. Most microfluidic chip processing and microfluidic operation are complicated, which is not conducive to its popularization and application. The microfluidic chip in this study is easy to operate and requires less amount of specimen. Here, it was found that, in the microfluidic chip device, protocatechin has an inhibitory effect on platelet aggregation induced by the shear-rate gradient, which is concentration-dependent within a certain concentration range. Therefore, we believe that microfluidic chip technology can inhibit platelet aggregation induced by a shear-rate gradient produced by protocatechuic acid as a new type of antiplatelet aggregation analysis technology for clinical use. This study mainly designed a simple microfluidic chip to analyze the inhibitory effect of protocatechuic acid on platelet aggregation, induced by gradient changes in shear rate in vitro, and promote the chip technology and screen for other potential antiplatelet aggregation medicine analysis.

## 2. Materials and Methods

### 2.1. Materials

Protocatechuic acid (content ≥97.0%, Aladdin), Sylgard 184 polydimethylsiloxane (Dow Corning, USA), calcein Am fluorescent dye (Invitrogen), CD42b antibody (Biyuntian Biotechnology Company), dimethyl sulfoxide (Biyuntian Biotechnology Company), acetylsalicylic acid (Biyuntian Biotechnology Company), tirofiban (Broad Pharmaceutical (China) Co., Ltd.), ristocetin (HYPHEN), ADP (Tali Kangxin), collagen (Tali Kangxin), kaolin activator (Haemoscope, USA), sodium citrate venous blood vacuum tube (Shandong Weigao), thromboelastometer (Haemoscope, USA), RSP01-CS two-way push-pull precision injection pump (Jiashan Ruichuang), IX71 inverted fluorescence microscope (Olympus), and plasma cleaner (PDG-32G-2) were used in this study.

### 2.2. Collection and Processing of Blood Samples

Venous blood samples were collected from 20 healthy volunteers after clinical examination recruited at the Physical Examination Center of Yong-Chuan Hospital, affiliated with Chongqing Medical University, between March and June 2019. Subject eligibility criteria were as follows: healthy volunteers who have not taken clopidogrel, aspirin, or other antiplatelet aggregation drugs and statins in the prior 2 weeks, and their platelet counts were within the normal range.

This study was approved by the Ethics Committee of Yong-Chuan Hospital (approval number 2018016), and all subjects provided written informed consent to participate in the study. The blood samples were stored in a vacuum blood collection tubes with 1 : 9 (v/v) 3.2% sodium citrate and used within 2 h of collection. Calcein AM was used to fluorescently label platelets in blood samples. Calcein AM, a fluorescent dye targeting viable cells, can penetrate the cell membrane and enter the cell after being cleaved by intracellular esterase to form calcein, which emits a strong green fluorescence. During the experiment, the calcein AM fluorescent dye at 1 mmol/L was added to the blood sample at a ratio of 1 : 500 (v/v), and the mixture was shaken gently and incubated at 37°C in the dark for 20 min.

### 2.3. Preparation of the Microfluidic Chip

**Figure 1:**
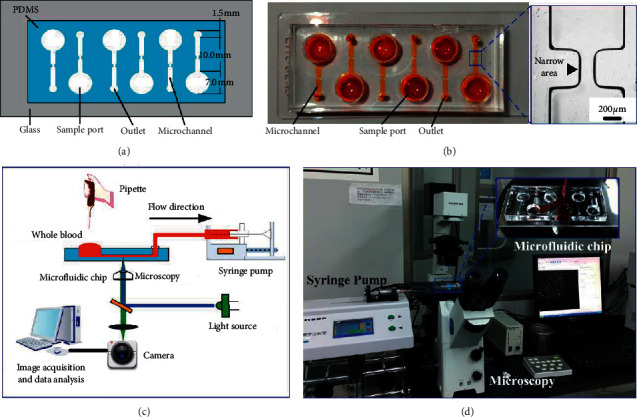
(a) Schematic diagram of the microfluidic chip structure. (b) Physical diagram of the microfluidic chip, with orange dye indicating the sample pool, microchannels, and outlets of the microfluidic chip. (c) A schematic diagram of the operation principle of the analysis system. (d) Physical analysis of the system.

The platelet adhesion aggregation analysis microfluidic chip designed in this study contains 80% fixed narrow microchannel. The chip was prepared as previously outlined [15]. The microfluidic chip designed in this study consists of a reservoir and a narrow microchannel. The liquid sample in the reservoir enters the microchannel under the negative pressure exerted by the outlet syringe. The physical map and schematic diagram of the microfluidic chip are shown in Figure 1. Each chip contains six, independent, parallel microchannels, so six experiments can be performed independently.

### 2.4. Microfluidic Chip Detection of Platelet Aggregation Behavior

Before using the microfluidic chip for analysis, the microchannel was subjected to hydrophilic treatment according to the following process. (1) Treat the microfluidic chip with a plasma cleaning machine (30W) for 2 min to increase the hydrophilicity of the microfluidic chip. (2) Place the treated microfluidic chip on the stage of an inverted fluorescence microscope and use PTFE; the vinyl tube (inner diameter, 1.0; outer diameter, 1.5 mm) connects the chip outlet to the syringe pump and uses the pull-back mode to control the flow rate of the blood sample in the microchannel. (3). When blood starts to flow in the microchannel, use the StreamPix 5.0 software to make the camera record images at a frame rate of 1 frame/s of the adhesion and aggregation of fluorescently labeled platelets on the downstream region of the narrow surface after flowing through the microchannel (objective lens, ×20); record for 5 min to obtain a total of 300 serial fluorescence images. The schematic diagram and photographs of the experimental device are shown in Figure 1. The flow rate in the narrow channel was set to 10 uL/min, 50 uL/min, and 100 uL/min. According to the finite element analysis, the three flow velocities can reach shear rates of 1000 s^−1^, 5000 s^−1^, and 10,000 s^−1^, respectively. The above three flow rates simulate the shear rate of the normal physiological flow rate, moderately narrowed blood vessels, and severely narrowed environments, respectively. The calcein AM fluorescent dye-labeled sodium citrate-anticoagulated whole blood samples were incubated at 37°C in the dark for 20 min and then added to the sample pool to observe the platelet aggregation behavior in real time.

### 2.5. Inhibitory Effects of Different Inhibitors on Platelet Aggregation

The microfluidic chip was used to detect the inhibitory effect of drugs on platelet aggregation. For these experiments, a mixture of the platelet inhibitor CD42b (200 *µ*g/mL), tirofiban hydrochloride (50 *µ*g/mL), and protocatechuic acid (concentrations of 1, 2, 4, 6, 8, and 16 *µ*g/mL) in 1 mL of sodium citrate-anticoagulated whole blood solution was incubated at 37°C for 20 min in the dark and subsequently added to the microfluidic chip sample pool to observe the platelet aggregation effect in real time. The experiment was repeated in triplicate.

### 2.6. Platelet Aggregation Function Test

A thromboelastometer and an automatic platelet aggregation instrument were used to detect the platelet aggregation function. In these experiments, 2 *µ*l of protocatechuic acid solution was added to 1 mL of whole blood solution and the mixture was incubated for 20 min; then, 340 mL of this mixture was added to the test cup of the elastic graph instrument to evaluate the inhibitory effect of protocatechuic acid on platelet aggregation. The test was repeated three times. Subsequently, the whole blood samples were centrifuged at 160 × g and 2,000 × g for 10 min each, and 900 *µ*l of platelet-rich plasma and 600 *µ*l of platelet-poor plasma were taken to test the platelet aggregation function and observe the inhibitory effect of protocatechin on platelet aggregation, respectively. The test was repeated three times.

### 2.7. Statistical Analysis

The recorded data were inputted into Microsoft Excel, and the statistical software, SPSS 19.0, was used for data analysis. Data are represented as the mean ± calibration error; comparison between groups was performed using one-way analysis of variance. *P* < 0.05 was considered as statistically significant.

## 3. Results

### 3.1. Shear-Rate Gradient Changes in the Microchannel Analysis Platform

**Figure 2:**
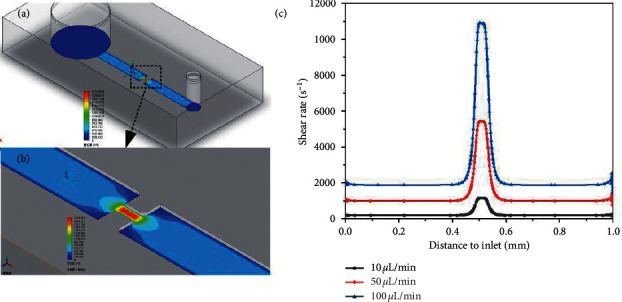
(a) Chromatic scale of the shear rate of a narrow microchannel. (b) Shear-rate distribution at the stenosis. (c) Shear-rate distribution in the narrow microchannel.

In this study, we used the finite element analysis module of the 3D modeling software solidwork to analyze the fluid dynamic behaviors in the chip (as shown in Figure 2). The shear-rate distribution in most areas of the narrow microchannel was uniform, and only two gradient areas near the stenosis are shown in Figure 1(a) and 1(b). As can be seen in Figure 1(c), the length of the narrow channel is 0.5 mm, the upstream and downstream shear-rate gradient change area is located at 0.75 mm before and after the narrow channel, and the gradient decreases from the narrow to both ends. When the flow rate is 100 *µ*l/min, the maximum shear rate in the channel can reach 11,000 s^−1^. When the narrow microchannel is close to the stenosis, the shear rate is high and the fluid velocity is fast. In contrast, when the shear rate is low, the fluid velocity is slow moving away from the constriction. Therefore, platelets will slowly aggregate at the place where the downstream flow slows, gradually forming stable platelet microaggregates. This has also been demonstrated in our previous experiments. Therefore, the platelet adhesion aggregation image studied in this study selected the aggregation area downstream of the stenosis at 0.75 mm.

**Figure 3:**
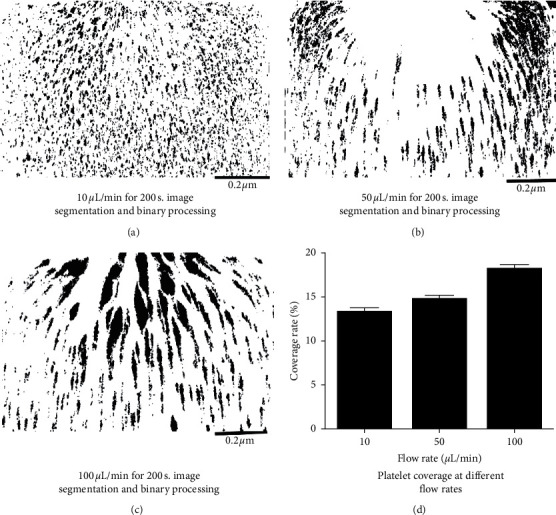
(a–c) Image analysis graphs of platelet coverage. (d) Bar graph of platelet surface coverage at different flow rates.

Passage of calcein AM-labeled whole blood through the microchannel at different flow rates showed that platelets had aggregated to different degrees in the channel. The image segmentation and binarization of platelets are shown in Figure 3. When the flow rate was 10 *µ*l/min, there was a 1,000 s^−1^ arterial physiologically relevant fluid shear rate condition in the narrow channel. After the blood passed through the narrow microchannel, small and weak fluorescence intensity was scattered on the surface of the microchannel, causing a uniform distribution of platelets; the coverage rate of microaggregates and platelet aggregates was 13.18 ± 0.138%. When the shear rate increased to 5,000 s^−1^, the area covered by platelet aggregates increased to 14.309 ± 0.325%, and when the shear rate increased to 10,000 s^−1^, the platelets downstream of the narrow channel aggregated into high-brightness clumps, indicative of platelet aggregates, and the coverage area was 17.271 ± 0.452%. This difference was statistically significant (*P* < 0.05; Figure 3(d)). In addition, the image shows that the greater the shear rate of the blood, the larger the platelet aggregates, but their density decreased, which is consistent with the results of other researchers. Chan et al. [16] analyzed the vWF polymer, and the results showed that as the shear rate increased, high-molecular weight vWF polymer was split into low-molecular weight vWF polymers and ADAMTS13 shear-mediated high-molecular weight vWF. The polymers were degraded simultaneously. In addition, in this study, blood was in a physiological flow state, and platelets had not yet formed stable aggregates downstream of the stenosis; therefore, some unstable aggregates may have been washed away.

**Figure 4:**
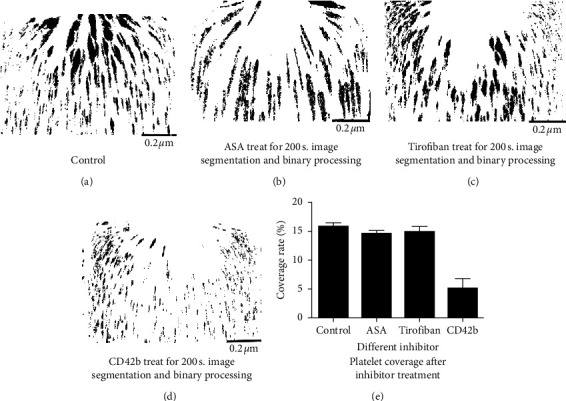
(a–d) Image analysis graph of platelet coverage. (e) Bar graph of platelet surface coverage after treatment with different inhibitors.

The passage of samples processed using acetylsalicylic acid (ASA, 200 *µ*mol/mL) and tirofiban hydrochloride (50 *µ*g/mL) through the microchannel and the image segmentation and binarization of platelet microaggregates aggregated downstream of the channel are shown in Figure 4; the platelet coverage values were 13.224 ± 0.351% and 14.856 ± 0.219%, respectively, and the difference was not statistically significant (*P* > 0.05). The platelet coverage rate of CD42b-treated samples through the microchannel was 5.477 ± 0.109% (*P* < 0.05).

**Figure 5:**
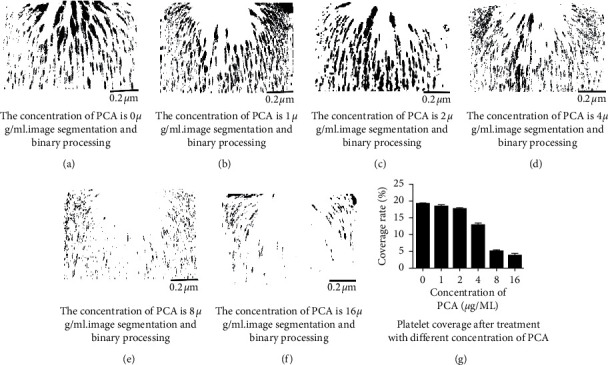
(a–f) Image analysis graphs of platelet coverage. (g) Histogram of platelet surface coverage at different concentrations of protocatechin.

**Figure 6:**
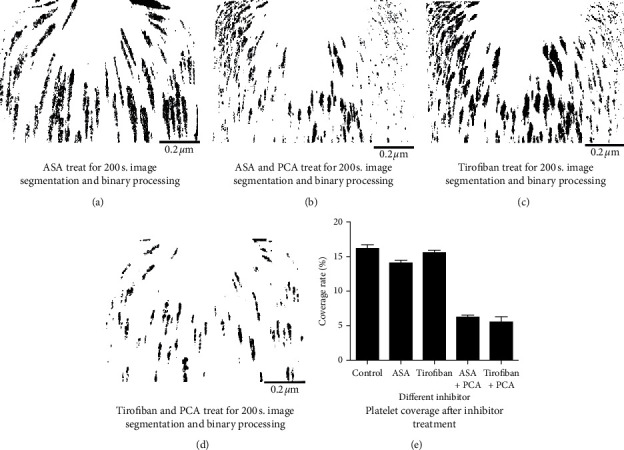
(a–d) Image analysis graphs of platelet coverage. (e) Histogram of platelet surface coverage at different concentrations of protocatechin.

The inhibitory effect of different concentrations of protocatechuic acid on platelet adhesion and aggregation was subsequently analyzed with calcein AM-labeled blood treated with different concentrations of protocatechin after passage through the microchannel (flow rate 100 uL/min) for 200 s. Thus, the inhibitory effect on platelet aggregation was shown to be different. The image segmentation and binarization of platelet aggregates are shown in Figure 5. As the concentration of protocatechuic acid increases, the fluorescence intensity and platelet coverage of microaggregates formed by platelets in the microchannel showed a downward trend and gradually decreased, indicating a concentration-dependent inhibitory effect. Under the conditions of a shear rate of 10,000 s^−1^, when the concentrations were 1 *µ*g/mL and 2 *µ*g/mL, the platelet coverage rates were 19.031 ± 0.981% and 18.384 ± 0.638%, respectively (*P* > 0.05), as shown in Figure 5(g). However, when the concentrations were set at 4, 8, and 16 *µ*g/mL, the platelet coverage rates were 12.072 ± 0.73%, 3.721 ± 0.764%, and 2.996 ± 0.568%, respectively (*P* < 0.05). In this study, protocatechuic acid had an inhibitory effect on platelet aggregation induced by the shear rate produced by the microchannel, and there was concentration dependence in the concentration range set in the experiment (0–16 *µ*g/mL). At 8 *µ*g/mL, we observed a significant inhibitory effect on aggregation; therefore, 8 *µ*g/mL of protocatechuic acid was selected as the concentration to be used for subsequent experiments. Aspirin and tirofiban were combined with protocatechuic acid. After the two samples passed through the microchannel, the fluorescence intensity of platelet aggregates was weakened, and the platelet coverage rate was reduced (Figure 6).

### 3.2. Platelet Aggregation Function Test

Results are given in Table 1. Under static conditions, there were no significant differences in the various indices of the thromboelastogram under various concentrations of protocatechuic acid. The platelet aggregation function test MA value was 63.5 ± 2.3 (*P* > 0.05); no concentration of protocatechin was observed to inhibit platelet aggregation. No effect on blood coagulation function of the drug was observed from the test results. In the determination of platelet function, the maximum aggregation rates of AA, ADP, and COL were 82.3 ± 3.4, 78.3 ± 2.5, and 80 ± 4.1, respectively, *P* > 0.05, which indicated no significant difference. The platelet aggregation induced by ristomycin was significantly reduced by 8 *µ*g/mL protocatechuic acid, with an aggregation rate of 25.5 ± 3.1, *P* < 0.05, which indicated the difference was statistically significant. Protocatechuic acid had no obvious inhibitory effects on platelet aggregation induced by AA, ADP, and COL.

## 4. Discussion

Many in vitro studies have indicated that platelet adhesion and aggregation and platelet-mediated thrombosis rely on fluid shear rate [17]. With the maturity of micro-nano processing technology, an increasing number of studies have shown that microfluidic chips have unique advantages in studying platelet adhesion and aggregation behavior in vitro [18–20], including precise control of the fluid shear environment, accurate simulation of local structural features that interact with blood vessel walls in vivo, the ability to perform whole blood analysis, and reduction in blood sample consumption. This study adopted a simple microfluidic chip technology, which was developed and verified in the early stages of this research, to analyze platelet adhesion and aggregation under a fluid shear environment [16]. In this study, a low-cost photosensitive dry film was used to avoid liquid photoresistance, which not only reduced the processing cost and difficulty of using microfluidic chips but also did not require additional clean space and large equipment, making it easier to be processed at ordinary biological laboratories. In the absence of any other professional equipment, the minimum width of the microchannel that can be processed by this method is 50 *µ*m, and the depth range is 30–200 *µ*m. In addition, the device is designed with a microchannel with a width of 1 mm and an 80% narrowness of 0.2 mm at the stenosis to observe the effect of protocatechuic acid on the adhesion and aggregation behavior of platelets in order to enhance the inhibition of platelet adhesion and aggregation by antiplatelet aggregation drugs in a flowing environment. The understanding of this behavior provides basic research data for the clinical application of microfluidic chips in the clinical diagnosis of platelet function and evaluation of the efficacy of antiplatelet aggregation drugs.

Compared with many microfluidic chips reported in the current research, the device studied in this research has certain advantages. First, the microfluidic chip structure, processing, and analysis process in previous reports have been relatively complicated, which limits the promotion and clinical application of microfluidic chip technology in platelet function analysis. Kim et al. [13] reported the antiplatelet aggregation effect of protocatechuic acid, but this study mainly used a cone and plate viscometer to shear platelets for a period of time to collect platelets to detect their aggregation by counting under an optical microscope. The results showed that a high-shear rate can induce platelet aggregation, but it cannot accurately reflect the effect of shear-gradient changes on platelet aggregation, and the platelet flow state could not be reflected during the experiment, which makes the operation more complicated. Therefore, the device in this study has been improved to detect platelet aggregation in a fluid state, with simple operation and a small number of specimens. According to the American CASS coronary artery disease vascular diagnostic criteria [21], in grade 0 (mild stenosis), the stenosis rate of the most severe blood vessel is <50%; in grade 1 (moderate stenosis), the stenosis rate of the most severe blood vessel is <70% but ≥50%; and in grade 2 (severe stenosis), the most severe, the blood vessel stenosis rate is ≥70%. Therefore, a blood vessel model that could represent 80% of severe stenosis was selected for this experiment. This device is set to narrow the two sides of the channel toward the middle, so that two symmetrical shear-rate gradient change regions appear before and after the narrowing. Therefore, this study found that protocatechuic acid could inhibit platelet aggregation induced by shear gradient in a concentration-dependent manner over a concentration range of 0–16 *µ*g/mL. Other antiplatelet aggregation drugs could not inhibit shear-induced aggregation.

Protocatechuic acid is a water-soluble phenolic acid component naturally present in many foods and traditional Chinese medicines [22–25], and it is the active substance of many traditional Chinese medicines, which has many beneficial effects, including potent antiplatelet aggregation, reduction of myocardial oxygen consumption, and improvement of myocardial pharmacological activities, such as increased oxygen tolerance, slowing down heart rate, bacteriostasis, and analgesia [26–28]. It also exerts antioxidant, antitumor, and neuroprotective effects [29, 30]. For example, studies have found that protocatechuic acid has anti-inflammatory, antibacterial [31, 32], antioxidant [33], and antitumor [34] effects and offers protection against cerebral hemorrhage [35]. Therefore, protocatechuic acid, an active ingredient in fruits and vegetables, has been studied for use in new drugs and will make a remarkable contribution to the future developments in medicine, and our research on protocatechuic acid will continue.

Studies have reported that protocatechin is highly selective in inhibiting platelet aggregation [13]. It exerts an obvious inhibitory effect on platelet aggregation caused by vascular stenosis but has no obvious inhibitory effect on platelet aggregation induced by the platelet activator ADP. Research data have shown that SIPA mainly depends on the interaction between plasma vWF and functional platelet receptor complex, GP1b/XI, but not on fibrinogen in plasma. However, in the normal circulatory system, vWF does not spontaneously bind to the GP Ib-IX-V complex. Only when shear stress occurs in the body, does vWF interact with the exposed subendothelial matrix. This may also be due to the induction of vWF A1 regional conformation changes [36]. In addition, in the liquid phase, the shear-dependent aggregation of platelets may also be triggered by the interaction between the vWF and GP Ib-IX-V complex. This process depends on the conformation changes of vWF and GP Ib-IX-V complex [37]. Among them, it is also related to the structure of GPIb itself. The N-terminal of GP Ib protrudes from the surface of the platelet membrane and is susceptible to shearing forces, which change the spatial configuration. Therefore, when subjected to high-shear forces, GPIb/IX, the vWF receptor on the platelet membrane, first binds to and promotes platelet aggregation to form a preliminary thrombus. We blocked the combination of GP1b/Xl and vWF by adding CD42b and found that platelet aggregation was significantly reduced, whereas addition of the GP IIb/IIIa receptor inhibitor tirofiban did not affect platelet aggregation. Therefore, we boldly speculate that the mechanism of protocatechuic acid on the platelet aggregation induced by shear rate may be a combination of GP1b/Xl or vWF blocking the interaction between GP1b/Xl and vWF. Second, it is also possible that the protective effect of protocatechuic acid on the structure of the platelet itself makes the platelet glycoprotein structure more stable, thereby preventing the high-shear rate from changing the structure of platelet GP Ib. Protocatechuic acid targets the platelet adhesion link; it inhibits platelet aggregation by antagonizing the interaction of the platelet membrane glycoprotein GPIb receptor and vWF. The platelet GP Ib-IX-V complex binds to the intima of exposed blood vessels. vWF induces pathological arterial thrombosis. Studies have shown that this initial step of blocking platelet adhesion reduces thrombus formation and reduces long-term restenosis of narrowed blood vessels after injury [36, 38].

In this study, we also used thromboelastography and photoelectric turbidimetry to detect platelet aggregation. Thromboelastography was first described by Dr. Harte in Germany in 1948. It is a quick cytology-based mode of coagulation detection using a thromboelastograph to describe the dynamic process of coagulation. Whole blood in microchannels simulates the entire process of blood coagulation in the body, enabling direct monitoring. This method makes up for the deficiencies of other traditional detection methods because it can detect coagulation activity in the state of platelet participation and can more truly reflect the coagulation status in the body. Thromboelastography uses whole blood to directly detect platelet aggregation function. The principle is to simulate slow venous blood flow in vitro. When whole blood is activated by an inducer, the blood coagulates into a thrombus. The sensor measures thrombus formation, and the blood coagulation speed and intensity curve are drawn by a computer. The advantages of this process are that it is simple, fast, easy to standardize and has good repeatability. It reflects the whole process of dynamic blood coagulation. The main influencing factors include interactions between blood components, such as platelets, coagulation factors, and fibrinogen, the speed of blood coagulation, and the level of activity of the fibrinolytic system. It is mainly used to guide clinicians' rational use of blood thinners. However, the disadvantage of this method lies in the simulation of slow venous blood flow during the detection process. The sample rotates back and forth at an angle of 4°45′, with a small amplitude and small shear force, which simulates platelet aggregation under physiological flow, but for pathological conditions, the blood flow speed cannot be achieved. Aggregation is a physiological characteristic of platelets, and it currently plays an important role in the processes of hemostasis and thrombosis. Commonly used methods to detect platelet aggregation function in the clinic include photoelectric turbidimetry and the electrical impedance method [39]. In the photoelectric turbidimetric method, an inducer is added to platelet-rich plasma (PRP) under specific continuous stirring conditions. As the platelets aggregate, the turbidity and transmittance of PRP change accordingly. When the platelets are fully aggregated, the light transmittance tends to be constant. The photodetector can receive the electrical signal of the continuous change in the light transmittance during the platelet aggregation reaction and convert it into a platelet aggregation curve. According to the curve, the degree of platelet aggregation is calculated, which is mainly used in the detection of antiplatelet aggregation drugs. However, in this study, the concentration-dependent inhibitory effect of protocatechuic acid on platelet aggregation could not be observed using the two detection methods. Therefore, the current traditional detection methods still have defects in detection. Most of them can only reflect the degree of platelet inhibition from one or several aspects and cannot reflect the true level of platelet function in the body.

In this study, a method to carry out simple experiments using microfluidic chip cell analysis in general biomedical laboratories was established to study the inhibitory effect of protocatechuic acid on SIPA. Compared with the advanced microfluidic cell analysis applications with multimodule integration reported in the literature, the microfluidic chip technology reported in this study is based more strongly on the practical application of ordinary biological laboratories, with simple operation, low cost, and easy repeatability of experiments. In addition, this method does not require expensive special equipment, mainly because it can simulate the real flow of blood in the blood vessel. Specifically, the microfluidic chip uses photosensitive dry film soft lithography technology, which uses photosensitive dry film instead of liquid photoresist to prepare the microfluidic chip male mold, which not only reduces the processing difficulty and cost but also reduces the processing requirements for experimental equipment and the environment; the use of microfluidic chip technology to monitor the aggregation effect of protocatechuic acid on platelets can make up for the defects of other detection methods. However, this study also has certain shortcomings. The experimental research involved is still at the stage of in vitro research. Next, we will conduct in vivo animal studies of drugs; second, we will further study the mechanism of drugs inhibiting platelet adhesion and aggregation. In summary, the microfluidic chip technology established in this study is simple to operate, requires only a small amount of specimen, and can truly reflect the situation in the body. It can be used as a new type of antiplatelet aggregation analysis technology for screening and studying other potential antiplatelet aggregation drugs.

## 5. Conclusions

The microfluidic chip analysis technology developed in this study can be used to study the effect of protocatechin in inhibiting platelet aggregation induced by shear rate in vitro. This technology is simple to operate and can be used as a new type of antiplatelet aggregation analysis technology for screening studies of novel potential antiplatelet aggregation drugs.

## Figures and Tables

**Table 1:** Thromboelastography and photoelectric turbidimetry to detect platelet aggregation function (*n* = 40).

Concentration of PCA (*µ*g/mL)	MA	*P*	Maximum aggregation rate (%)				*P*
			AA	ADP	COL	Ristomycin	
0	64.2 ± 3.1	0.281	80.5 ± 2.6	79.2 ± 3.4	82.4 ± 2.7	85.1 ± 4.8	0.572
8	63.5 ± 2.3	0.352	82.3 ± 3.4	78.3 ± 2.5	80 ± 4.1	25.5 ± 3.1	0.009

*P* < 0.05 indicates a statistically significant difference.

## Data Availability

The data used to support the findings of this study are available from the corresponding author upon request.
